# Human Cardiac-Mesenchymal Stem Cell-Like Cells, a Novel Cell Population with Therapeutic Potential

**DOI:** 10.1089/scd.2018.0170

**Published:** 2019-04-25

**Authors:** Rachel Oldershaw, W. Andrew Owens, Rachel Sutherland, Martin Linney, Rachel Liddle, Lissette Magana, Gendie E. Lash, Jason H. Gill, Gavin Richardson, Annette Meeson

**Affiliations:** ^1^ Department of Musculoskeletal Biology, Faculty of Health and Life Sciences, Institute of Ageing and Chronic Disease, University of Liverpool, Liverpool, United Kingdom.; ^2^ Institute of Genetic Medicine, Cardiovascular Research Centre, International Centre for Life, Newcastle University, Newcastle upon Tyne, United Kingdom.; ^3^ Department of Cardiothoracic Surgery, South Tees Hospitals NHS Foundation Trust, Middlesbrough, United Kingdom.; ^4^ Guangzhou Institute of Pediatrics, Guangzhou Women and Children's Medical Center, Guangzhou, China.; ^5^ The Faculty of Medical Sciences, School of Pharmacy, Northern Institute for Cancer Research (NICR), Newcastle University, Newcastle upon Tyne, United Kingdom.

**Keywords:** cardiac stem cells, cardiosphere-cardiac-derived cells, single cell

## Abstract

Cardiac stem/progenitors are being used in the clinic to treat patients with a range of cardiac pathologies. However, improvements in heart function following treatment have been reported to be variable, with some showing no response. This discrepancy in response remains unresolved. Mesenchymal stem cells (MSCs) have been highlighted as a regenerative tool as these cells display both immunomodulatory and proregenerative activities. The purpose of this study was to derive a cardiac MSC population to provide an alternative/support to current therapies. We derived human cardiac-mesenchymal stem cell-like cells (CMSCLC), so named as they share some MSC characteristics. However, CMSCLC lack the MSC trilineage differentiation capacity, being capable of only rare adipogenic differentiation and demonstrating low/no osteogenic or chondrogenic potential, a phenotype that may have advantages following transplantation. Furthermore, CMSCLC expressed low levels of p16, high levels of MHCI, and low levels of MHCII. A lack of senescent cells would also be advantageous for cells to be used therapeutically, as would the ability to modulate the immune response. Crucially, CMSCLC display a transcriptional profile that includes genes associated with cardioprotective/cardiobeneficial effects. CMSCLC are also secretory and multipotent, giving rise to cardiomyocytes and endothelial cells. Our findings support CMSCLC as a novel cell population suitable for use for transplantation.

## Introduction

Cellular strategies using bone marrow-derived cells or autologous cardiac-derived cells have translated into the clinical setting as potential therapies for the treatment of patients with heart failure [1–3]. Although these trials have demonstrated some benefits of these therapies, they are also not without limitations, with reports of only modest improvements in cardiac function in some patients and no improvements in others. The mechanisms behind the improvements that have occurred remain unclear, it has been postulated that these transplanted cells either contribute to regeneration themselves giving rise to new cardiomyocytes or that they secrete paracrine factors to support native cardiac cells, spared by injury, allowing these cells to recover and promote some level of cardiac regeneration. Regardless of the mechanism, a better understanding of these cells may lead to improvement in cardiac regeneration. However, the choice of the most appropriate cell population for this application is yet to be determined. Mesenchymal stem cells (MSCs) have been selected as a useful cell population for tissue regeneration as they have been reported to be safe, have immunomodulatory effects, and to secrete proregenerative factors [4–8]. Human subendocardial MSCs have been derived; this cell population was capable of expansion in vitro, expressed markers normally expressed by MSCs, and was capable of multilineage differentiation to osteoblasts, adipocytes, and chondrocytes. Moreover, these cells express cardiac transcription factors while lacking expression of markers associated with mature cardiomyocytes [9].

In this study, we aimed to determine if we could isolate a cardiac cell population using tissue that is normally discarded as surgical waste (and therefore not necessitating any additional invasive procedures), from which we could derive cells that were MSCs or had MSC-like qualities and might therefore make them a more optimal tool to study cardiac regeneration. We opted for specimens of right atrial appendage (RAA), which are excised during coronary artery bypass surgery. RAA has previously been described as having characteristics of both atrial and ventricular tissue [10] and has been used as a source of stem cells used in a clinical trial [1]. We isolated a stem cell population that we have termed cardiac-derived mesenchymal stem cell-like cells (CMSCLC) and compared these with cardiosphere-cardiac-derived cells (CS-CDCs) (also RAA derived) and to bone marrow-derived MSCs (BM-MSCs). We demonstrate that while these CMSCLC are similar to other reported MSC populations, for example, have an MSC-like morphology, they also differ as they are largely incapable of the trilineage differentiation normally displayed by MSCs. Some CMSCLC could be made to differentiate into cells of the adipogenic lineage, and while displaying the morphology of osteoblasts under osteogenic culture conditions, contributed to no or negligible matrix mineralization, and under chondrogenic culture conditions did not undergo chondrogenesis. This was in contrast to CS-CDCs that differentiated readily to the osteogenic lineage, while also only displaying a low level of adipogenic lineage differentiation potential. However, both CMSCLC and CS-CDCs under in vitro cardiac differentiation conditions displayed morphological changes indicative of a more mature cardiac lineage cell, while the CMSCLC also showed early striation formation. In addition, we show that some CMSCLC express the senescence marker p16. We also undertook transcriptional analysis of single CMSCLC from three individual human heart tissue donors and showed that they expressed at varying levels; genes associated with pluripotency, proliferation, migration, differentiation, endothelial cells, cardiogenesis, and cardioprotective factors. These data provide further support for results obtained using a range of other techniques used in this study. Taken together, our data suggest that these CMSCLC differ from CS-CDCs. CMSCLC have a more limited ability to differentiate to noncardiac cell lineages than CS-CDCs, but can be expanded in vitro while retaining cardiac and some MSC characteristics.

## Materials and Methods

### Study approval

All studies were performed according to the amended Declaration of Helsinki. All cardiac tissue samples were collected from consenting patients undergoing cardiac surgery at the James Cook University Hospital, Middlesbrough, United Kingdom. Approval for collection and use of tissue was given by the local ethics committee under REC number UKCRN ID: 20120092.

### Derivation and culture of CMSCLC

**FIG. 1.**
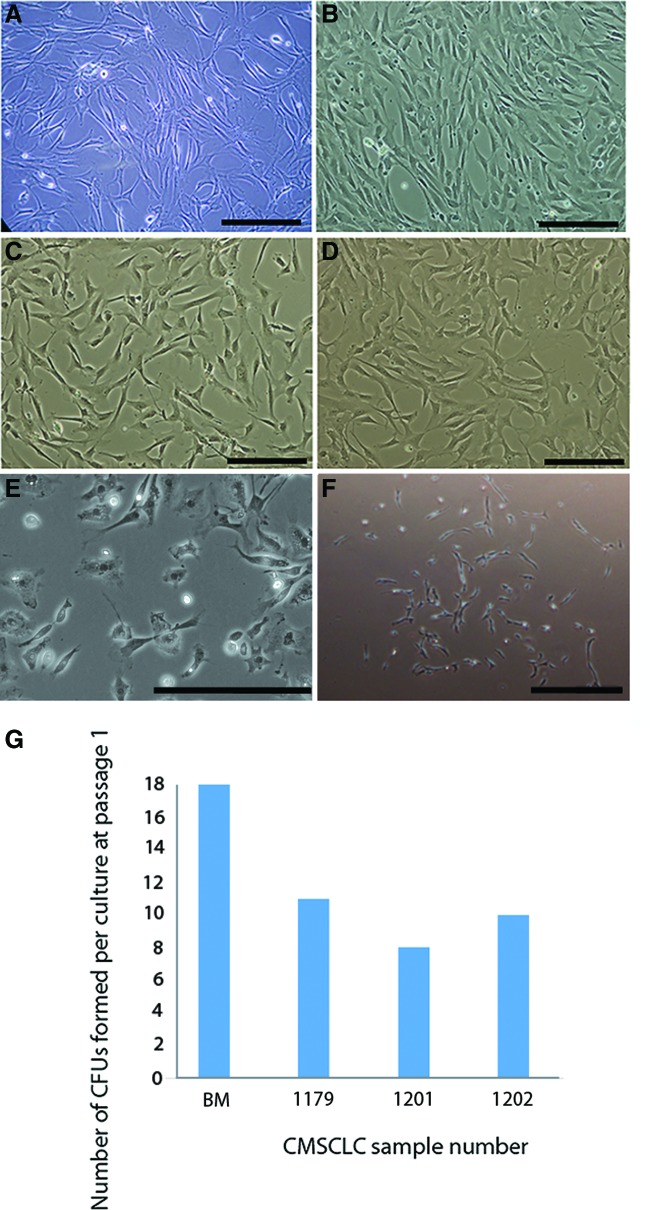
Morphology of different primary cells in culture. Representative brightfield images of cells in vitro at passage 3. **(A)** BM-derived MSCs, **(B–D)** show images from three different patient-derived cultures of CMSCLC. Brightfield image showing morphology of human CDCs **(E)** Brightfield image of CMSCLC CFU **(F)**. All scale bars = 100 μm. CMSCLC cultures derived from three different patient tissue samples were assessed for their ability to form CFUs under standard MSC culture conditions, and BM was also assessed for CFU formation ability **(G)**. The number of CFUs formed per culture at passage 1 is represented graphically. MSCs, mesenchymal stem cells; CMSCLC, cardiac-derived mesenchymal stem cell-like cells; BM, bone marrow. Color images are available online.

RAA [from five patients, three males—ages 62 (patient number 1,179), 66 (patient number 1,201), and 73 (patient number 1,201) and two females ages 66 (patient number 1,114) and 78 (patient number 1,132)], all undergoing coronary artery bypass surgery), was washed and chopped followed by digestion for 45 min in 0.01% (wt/vol) pronase (Calbiochem), during which the tissue suspension was rotated on an MACS rotator (Miltenyi) at 37°C and then tissue was dissociated using a gentleMACS dissociator (Miltenyi). The resulting cell suspension was then filtered through a 70 μm cell strainer (BD). Cardiac cell suspensions were made up to a total of 6 mL with MSC medium (αMEM 10% (vol/vol), fetal bovine serum 1% (vol/vol), GlutaMAX™ 5 ng/mL, and FGF2, (Peprotech), seeded into T-25 culture flasks, and cultured at 5% CO_2_, 5% O_2_. The culture medium was removed 72 h postseeding and nonadherent cells were removed by rinsing three times with Dulbecco's phosphate buffered saline (DPBS). Fresh culture medium was added and further medium changes were performed every three days thereafter. Colony-forming unit fibroblasts where defined as colonies if they contained 50 or more cells. Colony forming units (CFUs) were observed ∼15 days postseeding, and these ranged from 8 to 11 CFUs per CMSCLC culture and 18 CFUs for bone marrow (Fig. 1). All colonies from each individual donor were expanded as a pool until confluent. On confluence, cell cultures were expanded by passage at a ratio of 1:3 using TrypLE™ Express.

### Cell population doublings of CMSCLC

Cell population doubling of CMSCLC per day were calculated over three cell passages; the equation to calculate this is provided in the Statistical Analysis section and based on Ref. [11].

### Derivation and culture of cardiac-derived cells from cardiospheres

Cardiospheres were derived using methods described previously [12]. In brief, RAA (from three patients, one female age 84 and two males ages 59 and 65, all undergoing coronary artery bypass surgery) were minced into small <1 mm^3^ fragments in trypsin and transferred to fibronectin-coated plates in DMEM with 20% serum containing 0.1 mmol/l 2-mercaptoethanol (GIBCO). Following culture for 10 days, nonadherent-phase bright cells were harvested and cultured on poly-D-lysine in cardiosphere growing media. Several days later, cells that remained adherent to the poly-D-lysine-coated dishes were discarded, while detached cardiospheres were harvested and expanded as a monolayer of CDCs on fibronectin-coated plates.

### Derivation and culture of BM-MSCs

Bone marrow mononuclear cells (BMMNCs) were purchased from Lonza (27-year-old male donor). BMMNCs were resuspended in MSC medium, seeded at a density of 1.25 × 10^7^ cells/T-75 flask and cultured at 5% CO_2_, 5% O_2_. The culture medium was removed 72 h postseeding and nonadherent cells were removed by rinsing three times with DPBS. Fresh culture medium was added and further medium changes were performed every 3 days thereafter. On confluence, cell cultures were expanded by passage at a ratio of 1:3 using TrypLE Express.

### Immunophenotyping of MSCs

Cardiac cells were detached from tissue culture plastic using TrypLE Express and resuspended in flow cytometry (FACS) buffer to a cell density of 1 × 10^6^ cells/mL. Aliquots of 200 μL were transferred to 1.5 mL microfuge tubes and incubated at 4°C for 1 h with 5 μg/mL of primary antibody (CD44, CD90, CD105, CD106, CD146, CD166, CD19, CD45, or IgG isotype control). Primary antibodies and IgG control were taken from the Human Multipotent Mesenchymal Stromal Cell Marker Antibody Panel kit (R&D Systems). Cells were rinsed with phosphate buffered saline and then resuspended in FACS buffer with donkey anti-mouse secondary antibody conjugated to Alexa Flour^®^ 488 diluted 1:250 (Invitrogen). Cells were labeled with directly conjugated primary antibodies raised against MHC class I and MHC class II antigens (HLA-ABC-FITC, 5 μL of stock reagent in 200 μL of cell suspension, Beckman Coulter; PE mouse antihuman HLA-DR, 5 μL of stock reagent in 200 μL of cell suspension, BD) and c-kit (5 μg/mL of PE-conjugated primary antibody; BD). Controls where unstained CDC and CMSCLC from the same cell preparations. Analysis was done using FACSCanto II (BD) with a 488 laser and 530/30 emission filter, and data analysis collated using FACS DiVa software.

### Analysis of platelet derived growth factor-alpha expression by CMSCLC

Cells were prepared and analyzed as for immunophenotyping. A primary antibody used was 5 μg/mL of anti-human platelet derived growth factor (PDGFR)-alpha (R&D systems, MAB322) and secondary was donkey anti-mouse secondary antibody conjugated to Alexa Flour 488 diluted 1:250 (Invitrogen).

### Chondrogenic differentiation of cell populations

Chondrogenic differentiation of cell populations was performed as previously described [13], with slight modification. Briefly, cells were collected by enzymatic detachment from tissue culture plastic and centrifugation at 700*g* for 3 min. Cells were resuspended in chondrogenic medium at a cell density of 5 × 10^5^ cells/mL. Aliquots of 1 mL volume were dispensed into 15 mL conical tubes and cell aggregates formed by centrifugation at 700*g* for 3 min. The caps were loosened to allow for gas exchange and the cultures incubated at 5% CO_2_, 5% O_2_ for 14 days with medium changes every 2 days.

### Osteogenic differentiation of cell populations

Osteogenic differentiation of cell populations was performed as previously described [14]. Briefly, cells were seeded in MSC medium into 12-well tissue culture plates at a density of 2.5 × 10^3^ cells/cm^2^. Twenty-four hours postseeding, the medium was replaced with osteogenic medium. Cultures were maintained for 28 days at 5% CO_2_, 5% O_2_ with medium changes performed every 3–4 days.

### Adipogenic differentiation of cell populations

Adipogenic differentiation of cell populations was performed using the StemPro^®^ Adipogenesis Differentiation Kit (Gibco), as per the manufacturer's instructions; cultures were maintained under standard oxygen conditions for a total of 21 days.

### Histological evaluation of differentiated cell populations

Adipogenic cultures were evaluated by phase-contrast microscopy and adipogenic cells identified as cells with prominent clusters of cytoplasmic lipid vesicles at 21 days for cardiac cells, these were then stained with oil red O. Adipogenic cultures were incubated for 30 min at room temperature with oil red O (stock solution of 30% [vol/vol] oil red O in isopropanol diluted to 60% (vol/vol) in ddH_2_O). Excess oil red O solution was removed and the cultures rinsed with ddH_2_O. Osteogenic cultures were evaluated for matrix mineralization by alizarin red staining. Osteogenic cultures were incubated for 2 h at room temperature in 2% (wt/vol) alizarin red (pH 4.3 with 10% [vol/vol] ammonium hydroxide). Excess alizarin red solution was removed and the cultures rinsed extensively with DPBS to remove background staining. Chondrogenic cell aggregates were embedded in optimal cutting temperature compound cryopreservation medium and frozen on dry ice. Cryosections (7 μm) were cut onto slides for histological analysis of cartilage tissue formation. For safranin O staining, cell pellet sections were stained with Harris' hematoxylin for 4 min, destained in acid alcohol (1% vol/vol HCl, 70% vol/vol) for 10 s, and rinsed in deionized water. Sections were counterstained with 0.02% aqueous fast green FCF for 3 min, rinsed in 1% (vol/vol) acetic acid, and then stained with 0.1% aqueous safranin O for 5 min. The slides were rinsed, dehydrated, and mounted using DePeX mounting medium.

### Cardiac differentiation of cell populations

CS-CDCs and CMSCLC were seeded into 12-well tissue culture plates at a density of 2.5 × 10^3^cells/cm^2^ and placed under their respective culture conditions. After 3 days, the culture medium was replaced with cardiac differentiation medium (Cellutions) and this in turn was replaced every 4 days. After 7 days in cardiac differentiation medium, the differentiating CMSCLC cultures were transferred to incubation at 5% CO_2_, 22% O_2_ for a further 14 days of culture.

### Endothelial cell differentiation of CMSCLC

CMSCLC were derived as described above and then cultured in Endothelial Cell Growth Medium 2 (PromoCell) for 9 days under standard oxygen conditions, with medium being replaced every 3 days.

### Immunocytochemistry

Cardiac differentiated cells grown either on coverslips or in chamber slides were harvested after 2 or 3 weeks in cardiac differentiation media, rinsed with DPBS, and fixed in cold methanol at −20°C for 20 min. Primary antibodies used were cardiac troponin C 1:200 (Ab30807; Abcam), NXK2.5 1:200 (Ab35842; Abcam), alpha tropomyosin 1:200 (GTX113857; GeneTex), and cardiac actin 1:200 (GTX101876; GeneTex). The secondary antibodies used were donkey anti-goat AF488 (A-11055; Invitrogen), donkey anti-rabbit AF594 (ab150076; Invitrogen), and donkey anti-rabbit AF488 (A11008; Invitrogen). Negative controls were sections incubated as for primary staining but without the inclusion of primary antibodies. As a positive control, cells of the AC10 cell line (derived from adult human ventricular cardiomyocytes) [15] were also stained with the aforementioned antibodies. For confocal Z-stack imaging, a Nikon Eclipse Ti was used running NIS Elements AR 4.20.02 software.

Endothelial cell differentiation cells were grown in chamber slides for 9 days, medium removed, and cells fixed in ethanol for 10 min. Cells were immunostained for CD34 (1:250; M7165; DakoCytomation) using an avidin/biotin/peroxidase technique (Vectastain Elite ABC kit; Vector Laboratories) and the reaction developed for 1–2 min with 3,3′-diaminobenzidine (DAB; Sigma) containing 0.01% H_2_O_2_ to give a brown reaction product. Sections were lightly counterstained with Mayer's hematoxylin for 30 s, dehydrated, cleared in xylene, and mounted with DPX synthetic resin.

CMSCLC for senescence analysis at passage 3 were maintained under standard MSC culture conditions in chamber slides for 24 h and then fixed. Primary antibody used was p16 1:50 (J0411; Santa Cruz), and the secondary was donkey anti-rabbit IgG (H+L), Alexa Fluor 488 (A-21206; Thermo Fisher) (1/1,000). Under × 20 magnification, 5 distinct fields of cells were imaged using an Axio imager 2 microscope (Zeiss) and the percentage of positive p16 expressing cells determined from these images. ICC images to show p16 were obtained using a confocal Nikon Eclipse Ti using NIS Elements AR 4.20.02 software as mentioned above.

### In vitro protein expression quantification

Following staining with NKX2.5 and troponin C antibodies, cultures were imaged at random locations (*n* > 5). Using ImageJ software (ImageJ; U.S. National Institutes of Health; http://rsbweb.nih.gov/ij/), total cell numbers were counted based on DAPI-stained nuclei. The percentage of positive cells for each protein was calculated based on the number of cells labeled with the specific antibody as a percentage of the total cell numbers. All quantification was performed in a blinded manner.

### Polymerase chain reaction

Polymerase chain reaction (PCR) was performed as described previously [16], PCR was run for 25 and 35 cycles to ascertain that comparisons of expression were made at the exponential phase and not once cDNA amplification had plateaued, data are shown for 35 cycles. The primer sequences used were as follows: MEF2C forward: CGAGATACCCACAACACACG, reverse: CGCTTGACTGAGGGACTTTC; GATA4 forward: TCCCTCTTCCCTCCTCAAAT, reverse: TCAGCGTGTAAAGGCATCTG; and β-actin forward: GCGGGAAATCGTGCGTGAC, reverse: GGAAGGAAGGCTGGAAGAG.

### ELISA

Cell culture supernatants (control was MSC media only) from CMSCLC at day 14 and 17 of culture were analyzed for IL-10, VEGF, FGF2, and TGFβ1 secretion by ELISA, according to the manufacturer's instructions (IL-10, VEGF, FGF2, TGFβ1 DuoSet; R&D).

### Single-cell capture

Single cells were captured and the loci of interest preamplified using the C1 system (Fluidigm, software version 2.2.1) following the manufacturer's protocol. Cells were partitioned using a 10–17 μm C1 Single-Cell Preamp integrated fluidic circuit (IFC) (PN 100-5479; Fluidigm). IFC priming, cell loading and lysis, reverse transcription, and preamplification were then carried out using reagents from the following kits: C1 Single-Cell Auto Prep Reagent Kit (PN 100-5139; Fluidigm), Ambion Single Cell-to-CT qRT-PCR Kit (4458237; Thermo Fisher Scientific), and 20X TaqMan Gene Expression primers (Thermo Fisher Scientific). Amplicons were transferred from the IFC to a 96-well plate and stored at −20°C.

### Single-cell gene expression analysis

Single cell gene expression analysis was carried out using the BioMark HD system and IFC Controller HX (Fluidigm) as per the manufacturer's instructions. Gene expression analysis was carried out using Dynamic Array Flex Six Gene Expression IFCs (Fluidigm). Assays were run using reagents from the following kits: Flex Six Gene Expression Reagent Kit (Fluidigm), 20X TaqMan Gene Expression primers (Thermo Fisher Scientific), and TaqMan Fast Advanced Master Mix (Thermo Fisher Scientific). TaqMan primers used are listed in Supplementary Table S1.

### Analysis of single-cell data

CT values were calculated using the Fluidigm Real-time Analysis software. The remainder of the analysis was carried out using data analysis primarily in R (version 3.4.1) and the expression values extracted using SINGuLAR Analysis Toolset v3.6.2. Low-quality cells were excluded from the analysis if total expression per cell was less than 250, or if the number of detected genes per cell was less than 10. The filtered data were normalized using the computeSumFactors from the scran R package. The remainder of the analysis was carried out using R core functions.

### Statistical analysis

Cell population doubling of CMSCLC per day was calculated over three cell passages using the following equation [17]: population doublings per day = ln N/N_0_ x *t*^−1^. Where N_0_ = number of cells seeded, *N* = number of cells counted at passage, and *t*^−1^ = number of days to passage. The percentage of p16 expressing cells was determined using SPSS box plot analysis. Excel 2013 was used to generate bar chart for single-cell data.

## Results

### Morphology, CFU forming potential, and doubling time of CMSCLC

CMSCLC were examined to determine if they had MSC-like characteristics when cultured under standard MSC culture conditions. At passage 3, BM-MSCs were plastic adherent and displayed a distinct fibroblast-like morphology (Fig. 1A), and CMSCLC also displayed these characteristics at passage 3 (Fig. 1B–D). The morphology of CS-CDCs is also shown as a comparison with the CMSCLC (1E). Colony forming potential is a recognized stem cell characteristic, an example of a CFU is shown (Fig. 1F). CMSCLC from three different patient-derived cultures readily formed similar numbers of CFUs around day 15 postseeding into culture (Fig. 1G) as control BM-MSCs were also used and formed CFUs at day 7 postseeding in vitro, CFUs were counted at day 15 (Fig. 1G). Cell doubling rates of CMSCLC were also determined for all three patient-derived cell cultures and no significant difference in doubling rates was observed (Supplementary Fig. S1).

### Phenotyping of CMSCLC

**FIG. 2.**
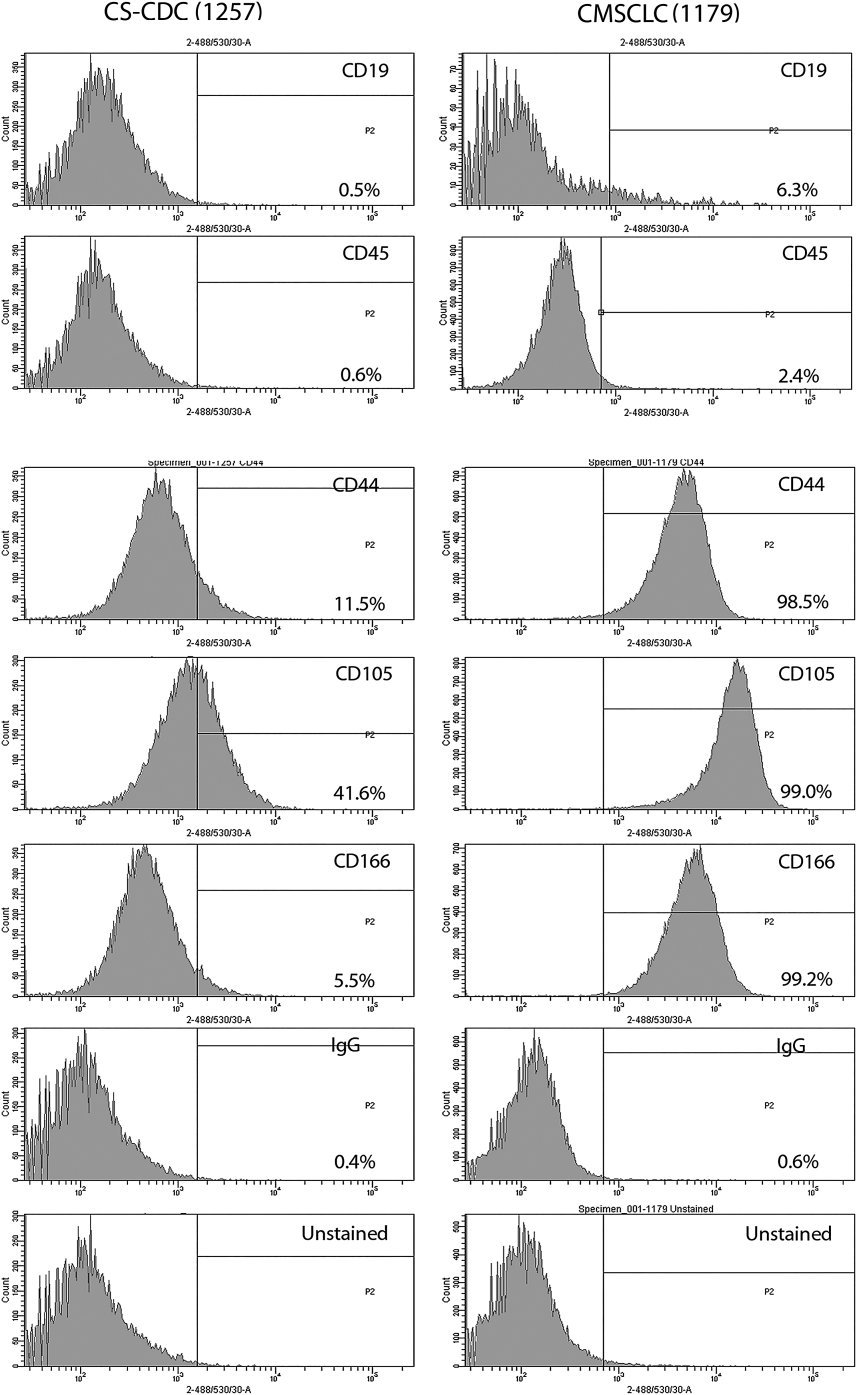
Immunophenotyping of CS-CDCs and CMSCLC. Representative images of FACS histograms showing results of immunophenotyping of CDCs (*n* = 3) and CMSCLC (*n* = 3) using cell surface antigens of both hematopoietic lineage-committed cells (CD45 and CD19) and those known to be expressed by MSCs (CD44, CD105, CD166). Controls are isotype control and unstained cells for both cell populations. All cell cultures were immunophenotyped at passage 3. CS-CDCs, cardiosphere-cardiac-derived cells; FACS, flow cytometry.

**FIG. 3.**
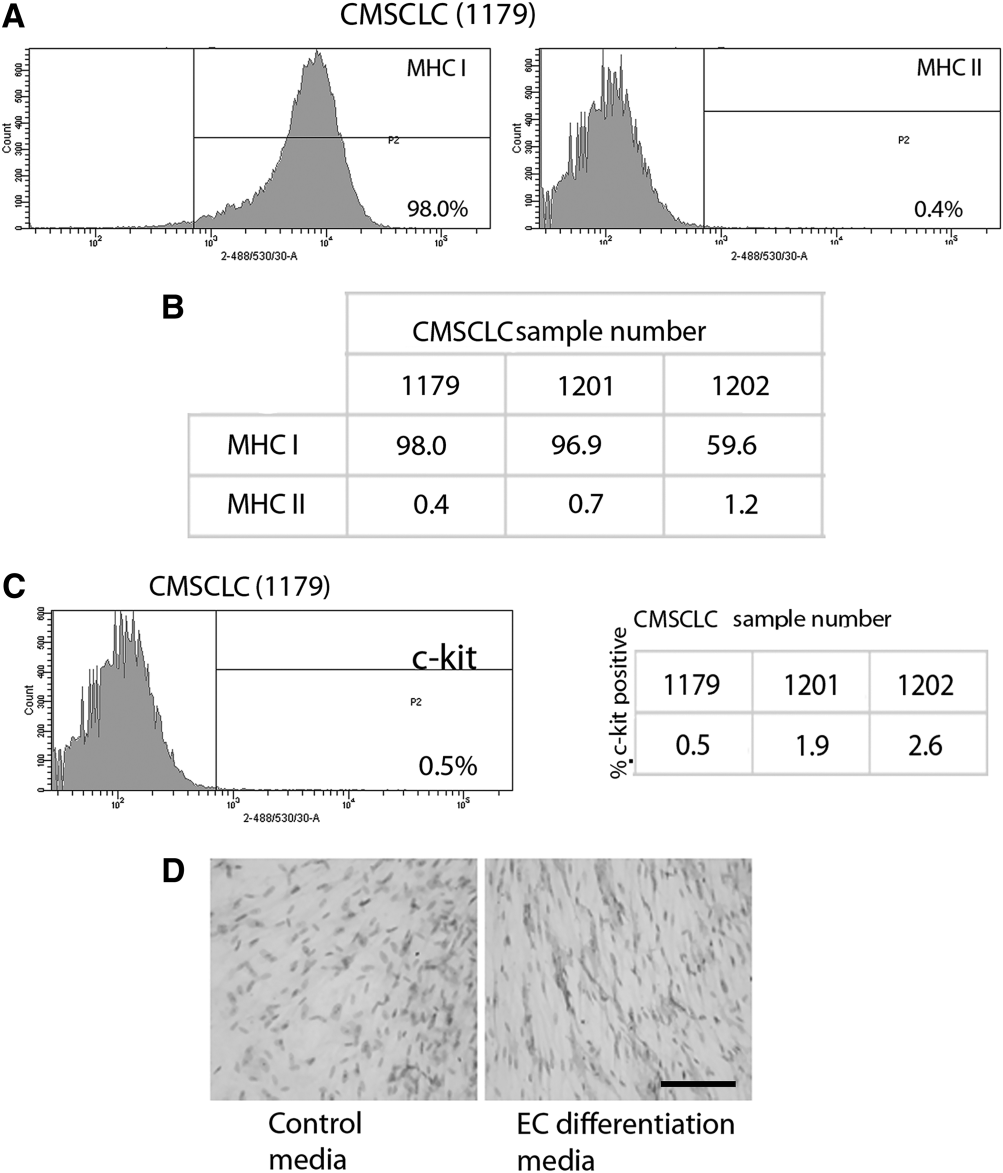
FACS analysis for expression of MHCs and c-kit, and IHC analysis for CD34 expression, by CMSCLC. **(A)** Representative images of FACS histograms showing expression levels of MHC I and MHC II by CMSCLC. **(B)** Table showing percentage of cells expressing MHCI and MHCII in three different patient-derived CMSCLC cultures. **(C)** Representative image of FACS histogram showing expression level of c-kit in CMSCLC and table showing percentage of cells expressing c-kit in three different patient-derived CMSCLC cultures. **(D)** Cells from the same patient-derived CMSCLC line were cultured for 9 days under normal CMSCLC culture conditions (Control) and under endothelial cell (EC) culture conditions (EC differentiation media) and were analyzed for the expression of CD34. Note lack of CD34 in cells cultured under normal CMSCLC culture conditions (magnification ×400). Scale bars = 100 μm. All cultures were examined at passage 3. IHC, immunohistochemistry.

CDCs and CMSCLC were screened for expression of cell surface antigens known to be expressed by MSCs. Both cell populations expressed CD44, CD105, and CD166, but the CDCs express all these markers at much lower levels than the CMSCLC (Fig. 2), while both expressed low levels of the hematopoietic lineage markers CD19 and CD45 (Fig. 2). Controls included IgG isotype control and unstained CDCs and CMSCLC (Fig. 2). CMSCLC were also tested for the expression of MHC I and II. Representative images of the data generated are shown (Fig. 3A), demonstrating that the majority of cells expressed MHC I (98%) and lacked expression of MHC II (0.4%). Controls included IgG isotype control-stained and unstained CMSCLC (data not shown). Three different patient-derived CMSCLC cultures were analyzed for MHC expression and the percentages of positive cells per culture are shown (Fig. 3B). CMSCLC were also examined for expression of c-kit, and this analysis revealed the presence of c-kit expression in only a rare subpopulation of cells (0.5%–2.6%) (Fig. 3C and Table). Controls were CMSCLC stained with IgG isotype control only and unstained cells (data not shown). In addition to immunophenotyping, the CMSCLC they were also immunostained (after 9 days in culture) for the expression of CD34. Cells from the same donor-derived CMSCLC line cultured under normal CMSCLC culture conditions showed no/low CD34 expression, while cells cultured under endothelial cell differentiation conditions showed increased expression of CD34 (Fig. 3D). We also examined CMSCLC for expression of PDGFR-alpha and showed that some cells express this marker (Supplementary Fig. S2).

### Secretion of IL-10, VEGF, FGF2, and TGFβ1 by CMSCLC

To determine if CMSCLC secreted IL-10, VEGF, FGF2, and TGFβ1, CMSCLC were cultured under normal CMSCLC conditions as described above and media were collected at day 14 and 17 of culture. In all cases, there was an increase in fold expression when compared with control media. After 14 days of culture, there was a 1.3-fold increase in IL-10 secretion, but by day 17, there was a 4.5-fold increase in IL-10 secretion. For VEGF at day 14, there was a 13.5-fold increase in VEGF secretion, which dropped to an 11.7-fold increase at day 17 compared with control, whereas for FGF2, at both day 14 and 17 there was an 8.9-fold increase compared with control. For TGFβ1, there was a 1.5-fold increase at both time points compared with control (Supplementary Fig. S3A). In all cases except for IL10 at day 14, the increase in expression compared with control was significant (Supplementary Fig. S3B).

### Osteogenic, adipogenic, and chondrogenic differentiation potential of CMSCLC

**FIG. 4.**
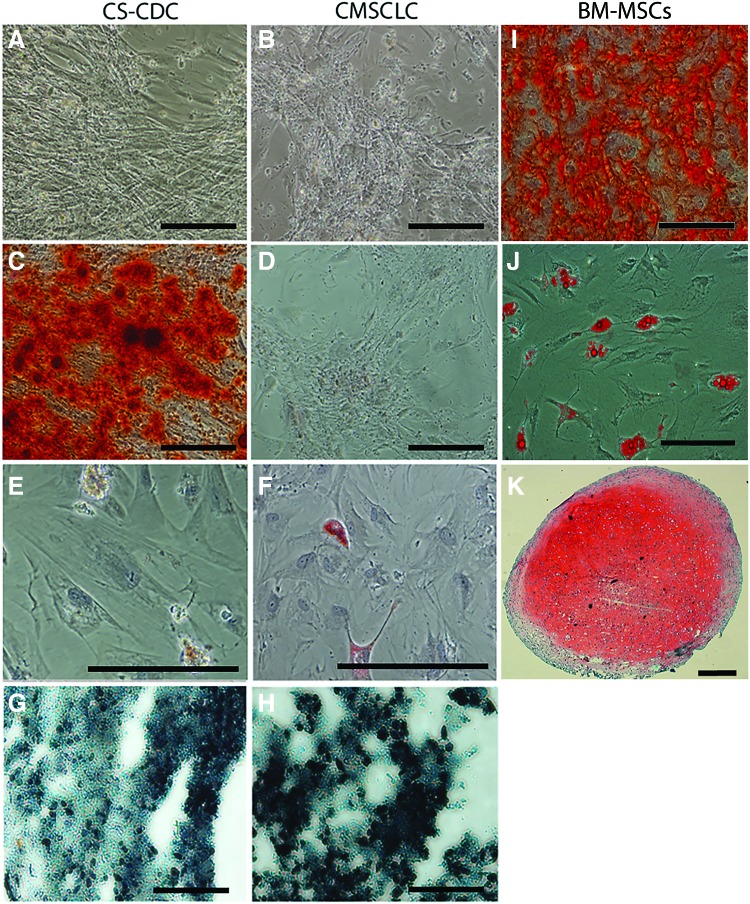
Characterization of trilineage differentiation potential of CDCs and CMSCLC at passage 3. Representative brightfield images, from three different patient tissue samples of **(A)** CDC and **(B)** CMSCLC after 28 days in osteogenic culture conditions and before alizarin red staining, demonstrate cellular proliferation and matrix deposition. Images of cells from the same samples but after being stained with alizarin red staining reveal mineralization of osteoid matrix within osteogenic cultures differentiated from CDCs **(C)**, whereas **(D)** there is no mineralization of culture matrix when CMSCLC were differentiated. Images of CDCs **(E)** and CMSCLC **(F)** after 21 days under adipogenic culture conditions stained with oil red O. Note the presence of red stained cells in both cultures. Images of cryosections of cell aggregates formed under prochondrogenic culture conditions after 14 days and after staining with safranin O (counterstained with hematoxylin and fast *green*) for CDCs **(G)** and CMSCLC **(H)**. Note that while cell aggregates formed for both CDCs and CMSCLC, chondrogenesis did not occur as evidenced by the absence of safranin O staining for sulfated glycosaminoglycans. Histological evaluation of cell aggregates shows lack of extracellular matrix deposition between cells and hence weak tissue structure. All scale bars = 100 μm. BM-MSCs are shown after culture using the same differentiation protocols as for CDCs and CMSCLC and stained with alizarin red **(I)**, oil red O **(J)**, and safranin O **(K)**. Note BM-MSCs can differentiate to all three lineages. Scale bars for **(I, J)** = 100 μm. Scale bar for **(K)** = 400 μm. BM-MSCs, bone marrow-derived MSCs. Color images are available online.

To determine the differentiation potential to the abovementioned lineages, CMSCLC, CDCs, and BM-MSCs were cultured under the appropriate lineage differentiation conditions. Cultures were examined microscopically for morphological changes, both CDCs (Fig. 4A) and CMSCLC (Fig. 4B) underwent morphological changes when cultured for 28 days under osteogenic culture conditions, but when stained for matrix mineralization using alizarin red only, the CDC cultures stained (Fig. 4C), whereas the CMSCLC showed low/no positive staining (Fig. 4D). Adipogenic differentiation was determined by the presence of cells with prominent clusters of cytoplasmic lipid vesicles that stained positively with oil red O after 21 days in culture (Fig. 4E, F). When cultured under chondrogenic culture conditions at 14 days, the CDCs (Fig. 4G) and CMSCLC (Fig. 4H) formed aggregates, but when evaluated histologically failed to stain with safranin O and had not undergone chondrogenic differentiation. BM-MSCs cultured under the same trilineage differentiation culture conditions provided a control and successfully differentiated to the osteogenic lineage and displayed matrix mineralization as indicated by alizarin red staining (4I), the adipogenic lineage as indicated by oil red O staining of cells containing cytoplasmic lipid vesicles (4J), and to the chondrogenic lineage as shown by staining of aggregates with safranin O (4K).

### Expression of p16 by CMSCLC

**FIG. 5.**
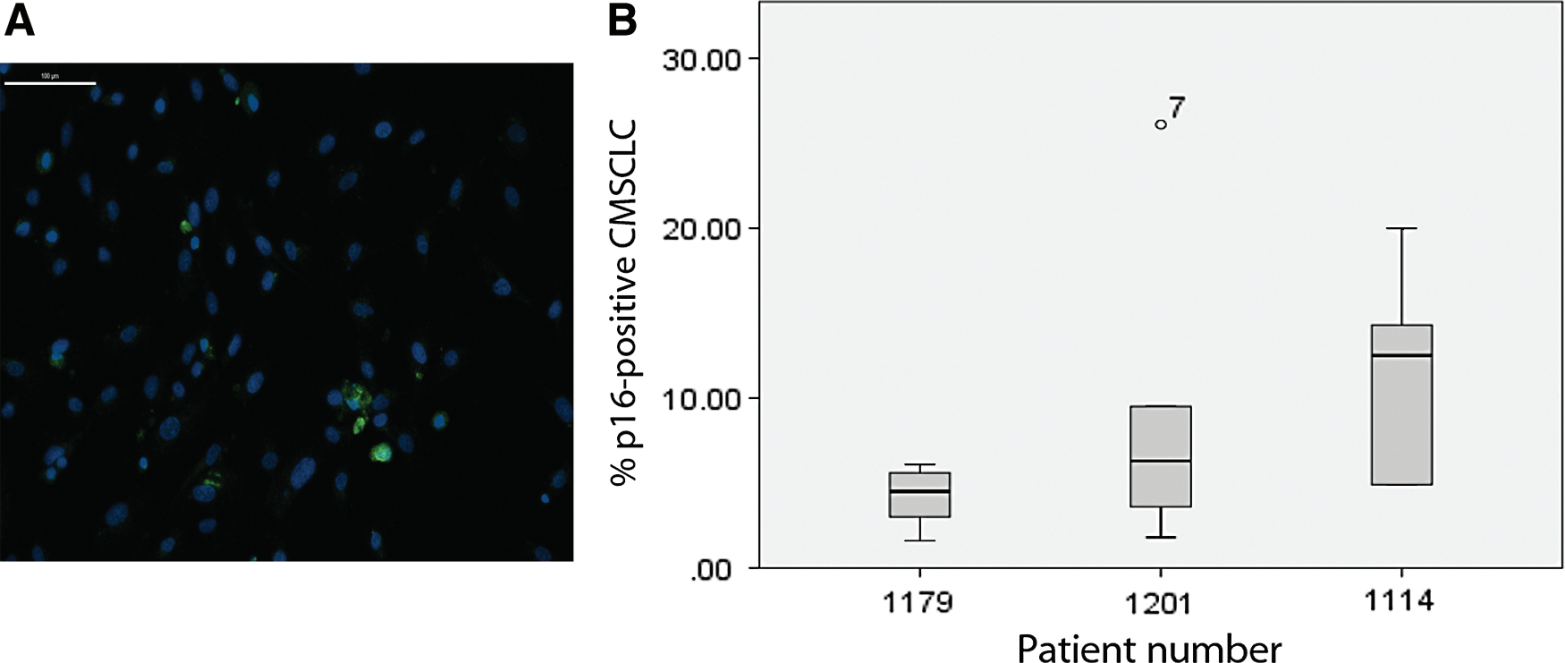
Quantification of CMSCLC p16 immunostaining. Representative image of p16 ICC showing results of staining of CMSCLC from patients, 1179, p16 (*green*), all nuclei stained with DAPI (*blue*), scale bar = 100 μm **(A)**. CMSCLC cultures derived from three different patient tissue samples were immunostained for the cell senescence marker p16 and counterstained with DAPI. The percentage of p16-positive cells within each culture was calculated by counting five independent fields within each culture. Box-plot analysis (SPSS) showed no significant difference in p16 expression between *n* = 3 patient cultures **(B)**. Color images are available online.

To establish if the level of senescence was higher in some patients' CMSCLC than others, we compared the level of senescence from CMSCLC at passage 3 from three different patients. The cultures were stained using p16 antisera. A representative image of p16-stained cells in a CMSCLC culture is shown (Fig. 5A). All cultures contained low percentages of p16 expressing cells (Fig. 5B).

### Cardiac differentiation potential of CMSCLC

**FIG. 6.**
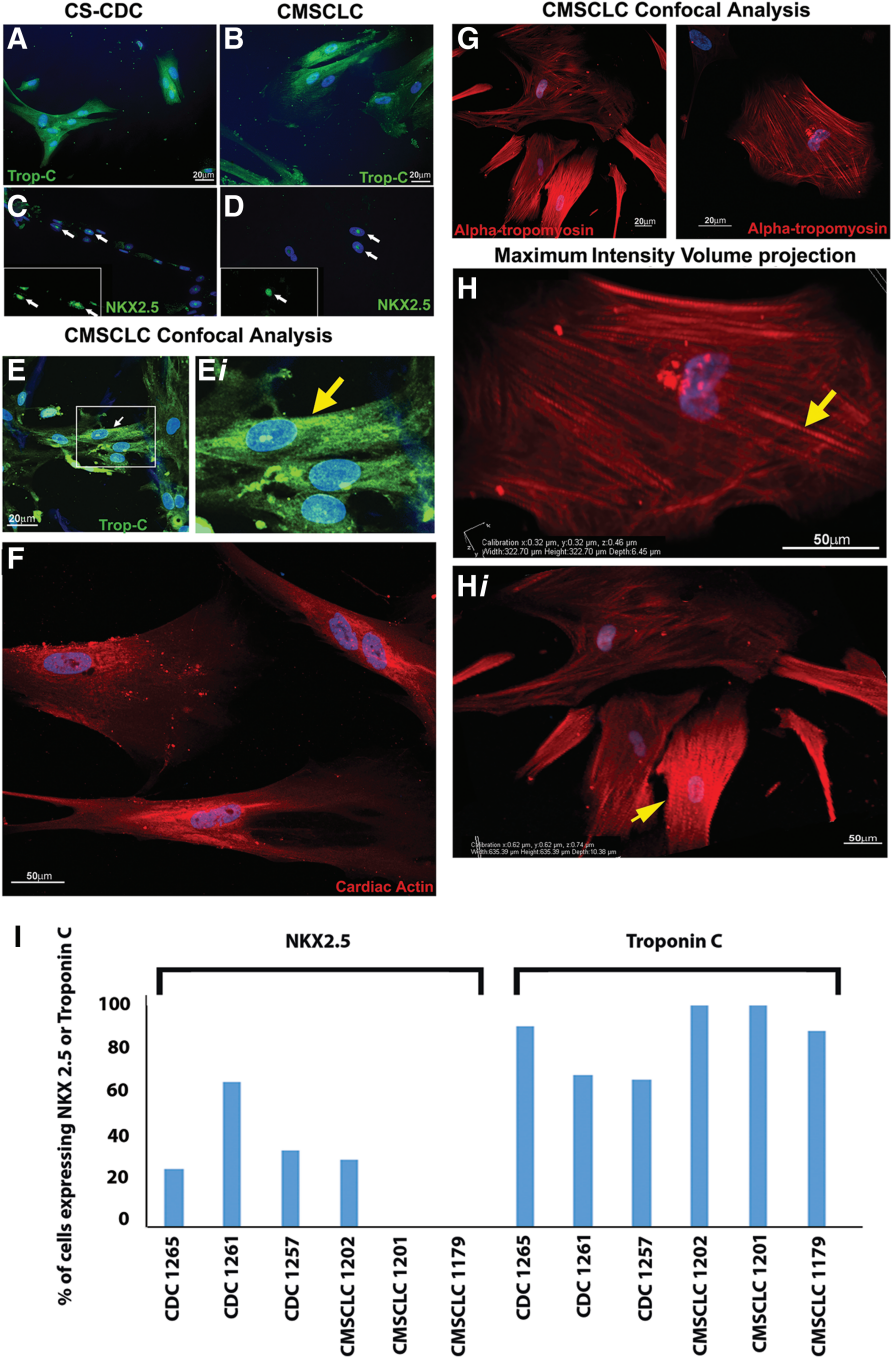
Characterization of cardiac differentiation potential of CDCs and CMSCLC. Representative images of CDC (*n* = 3 donors) and CMSCLC (*n* = 4) donors at 14 days under cardiac differentiation culture conditions show that some of both CDC **(A)** and **(B)** CMSCLC differentiated cells have a more mature cardiac morphology and express troponin C (*green*-Alexa Fluor 488). While other cells from the same patient samples cultured under the same conditions but stained for expression of NKX2.5 have a more primitive rounded morphology and express nuclear NKX2.5 (NKX2.5 *green*-Alexa Fluor 488) in differentiated CDC **(C)** and differentiated CMSCLC culture **(D)**. *White arrows* in **(C, D)** indicate cells shown in *inserts* of NKX2.5 staining without the DAPI overlay to shown the nuclear localization of NKX2.5. **(E–H)** Representative images of CMSCLC (*n* = 4 donors) differentiated for 3 weeks and analyzed by confocal microscopy. **(E)** Maximum intensity projections of differentiated CMSCLC expressing troponin C (*green*-Alexa Fluor 488). **(E*i*)** Higher magnification image demonstrates early striations, indicated by *yellow arrow*. **(F)** Maximum intensity volume projection of differentiated CMSCLC labeled with cardiac actin (*red*-Alexa Fluor 594). **(G)** Maximum intensity volume projection of differentiated CMSCLC labeled with alpha-tropomyosin (*red*-Alexa Fluor 594). **(H, H*i*)** Maximum intensity volume projection demonstrates striated pattern of alpha-tropomyosin expression, indicated by *yellow arrows*. For all images, nuclei labeled with DAPI-*blue*. Scale bars were either 20 or 50 μm as indicated. **(I)** Bar chart showing percentage of cells expressing NXK2.5 or troponin C, *n* = 3 for both CDC and CMSCLC cultures analyzed. Color images are available online.

To determine the ability of CMSCLC to differentiate to a more mature cardiac lineage, cells were cultured under cardiac differentiation conditions; after 2 weeks, the cells were stained for cardiac troponin I and NXK2.5 expression. CDCs have a previously demonstrated cardiogenic potential and were used as a positive control, being cultured under the same cardiac differentiation conditions for the same length of time. For both the CDCs (Fig. 6A) and CMSCLCs (Fig. 6B) for all cultures examined, troponin C expressing cells with a flattened, spread, and straight-edged morphology were present. While in all of CDC cultures and in one CMSCLC culture, some cells were present that expressed nuclear NKX2.5. Representative images of NKX2.5-positive cells are shown in CDC-derived cultures (Fig. 6C) and from the CMSCLC culture (Fig. 6D). The morphology of these cells differed from those shown (Fig. 6A, B), as these cells had a more primitive rounded morphology. To further demonstrate cardiomyocyte differentiation, CMSCLC were differentiated for 3 weeks, labeled for troponin C, cardiac actin, or the striated muscle-specific isoform of tropomyosin, α-tropomyosin, and imaged using Z-stack confocal microscopy. Maximum-intensity z-stack projections demonstrated filamentous staining for all proteins (Fig. 6E–H). In addition, maximum intensity volume projection of alpha-tropomyosin labeling clearly demonstrates the striated phenotype of these differentiating cells (Fig. 6H, H*i*). Note the absence of staining in negative controls (Supplementary Fig. S4A–C) and staining with all antibodies of cells of the AC10 cell line (Supplementary Fig. S4D). Analysis for percentage of cells expressing NKX2.5 and troponin C shows that the percentage of NKX2.5 expressing cells is lower than that of cells expressing troponin C for both cell populations, with the percentages of cells expressing troponin C being higher in all cases for CMSCLC (98%, 99%, and 84%) compared with CDCs (86%, 68%, and 63%) (Fig. 6I).

Molecular analysis was used to examine the undifferentiated CMSCLC and after culture under cardiac differentiation conditions for expression of genes important for cardiogenesis, both populations expressed MEF2C and GATA4 (Supplementary Fig. S5).

### Single-cell transcriptomic analysis

**FIG. 7.**
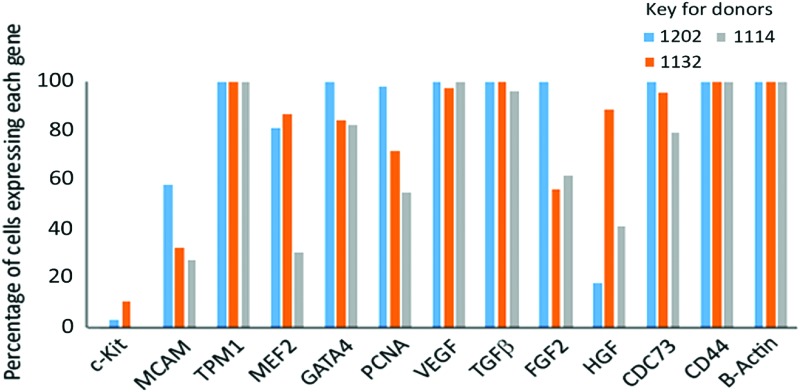
Graphical representation of single-cell data representing percentage of CMSCLC expressing genes of interest for *n* = 3 donors. The percentage of cell expressing each gene is shown. Color images are available online.

We examined CMSCLC derived from three patients; only cells that passed the quality control steps (see the Materials and Methods section) were included in the downstream analysis. The percentage of genes being expressed by cells in each culture was calculated (Fig. 7). B-actin was used as a housekeeping gene and was expressed by all cells. CD44 and tropomyosin were also expressed by all the cells analyzed. GATA4 (percentage of cells expressing ranging from 82.5% to 100%), VEGF (percentage of cells expressing ranging from 97.8% to 100%), and TGF-β (percentage of cells expressing ranging from 96.5% to 100%) were also highly expressed. CDC73 was more variable, being expressed by 100% of the cells in one culture and by 95.6% and 79.3% of cells in the other two cultures. The expression of FGF-2 (100, 56.5, and 62%), HGF (18.5%, 89.1%, 41.3%), PCNA (98.5%, 71.7%, 55.1%), MEF2 (81.5%, 86.9%, 31%), and MCAM (58.4%, 32.6%, 27.5%) also varied between the different donor cultures. c-Kit expression was also variable between the different donor-derived cultures with percentage of cells expressing being 3% and 10.8%, and with one culture having no detectable expression (Fig. 7). No expression was observed for NXK.25 in any of the cells of the three donor cultures analyzed (data not shown).

## Discussion

The International Society for Cell Therapy position article (2006) set the minimum requirements for cells to be considered MSCs as follows: the ability to adhere to plastic under standard culture conditions, to express a number of cell surface antigens, to lack expression of a number of markers, including the hematopoietic lineage markers CD45 and CD19, and to differentiate to osteoblasts, adipocytes, and chondroblasts in culture [18]. This article included the caveat that these criteria would need modification as our knowledge of MSCs increased. MSCs also have distinct cell morphology in vitro displaying a fibroblast-like morphology with cells showing parallel alignment and tapering of the fibroblast-like cellular protrusions [19]. CMSCLC displayed some of these characteristics, being plastic adherent in MSC culture conditions, having an MSC-like morphology, and expressing MSC markers CD105, CD166, and CD44 [20]. CMSCLC differed from CS-CDCs, displaying higher levels of expression of these antigens. Although controversial, human MSCs have been reported as being MHC I positive and having low/no expression of MHC II [21]. CDCs also express MHC I and lack expression of MHC II [22], and CMSCLC express MHC I and have low expression of MHC II. MSCs have been reported to have immunomodulatory properties, in part, due to their ability to secrete the anti-inflammatory cytokine IL10, which has been suggested to be cardiobeneficial. In a rat model of myocardial infarction, transplanted MSCs help reduce the inflammatory response and this was, in part, due to increased levels of IL10 [23]. We examined CMSCLC for IL10 expression and observed increased expression when cells where cultured for 14–17 days. In contrast to CS-CDCs, CMSCLC were unable to differentiate to the osteogenic lineage as they exhibit little/no matrix mineralization as indicated by the low/absence of alizarin red staining, while all CS-CDCs stained strongly for alizarin red. However, both cell populations exhibited low adipogenic differentiation and both failed to undergo chondrogenesis. This contrasts with BM-MSCs and subendocardial MSCs that have this trilineage differentiation potential [9]. These differences might be due to these being distinct cell populations, differences in tissue used for cell derivation (subendocardial MSCs are derived from ventricle, while CMSCLC are derived from atrial tissue), or due to differences in culture conditions used. CDCs and subendocardial MSCs are grown out from tissue explants, whereas CMSCLC are derived from single-cell suspensions and CMSCLC are cultured in 5% CO_2_, 5% O_2_, whereas no information is provided on the oxygen conditions for the culture of subendocardial MSCs. We have reported on increased derivation rates, osteogenic potential, and cellular health of MSCs derived from hemarthrosis fluid cultured under low oxygen conditions versus those cultured under standard conditions [14], supporting the hypothesis that culture conditions may influence cell behavior or possibly support the preferential selection of a subset of cells within a heterogeneous cell population. Certain cardiac stem/progenitor cells have been shown to be preferentially located in distinct anatomical regions of the heart, for example, adult human cardiac side population cells have been reported to be confined to the left atrium and absent from the right [24]. In a molecular study of multiple mouse cardiac stem cell populations, a comparison was made between the mouse data and microarray data available in the public domain and CS-CDCs appeared to be closely related to BM-MSCs [25]. This might also account for the osteogenic response displayed by our CS-CDCs. In addition, subendocardial MSCs were reported as being strongly positive for c-kit expression [9] and human CS-CDCs have been reported to express c-kit (about 18% of the total cell population) [26], while CMSCLC showed only low-level expression of c-kit. Identification of cardiac stem cells based on expression of c-kit alone has been brought into question. However, in a recent study, it has been reported that within the CD45^neg^c-kit^pos^ cells found in the heart, a small subpopulation of between 1% and 2% have the characteristics of true multipotent cardiac stem cells [17]. Therefore, the small percentage of c-kit expressing cells observed in CMSCLC cultures could represent such a population. The CMSCLC containing the highest percentage of c-kit expressing cells (2.6%) was also the only culture containing NkX2.5 expressing cells. PDGFR-alpha has been reported to be expressed by progenitor cells in human fetal and diseased adult heart [27]. Therefore, we also examined CMSCLC and observed that some expressed PDGFR-alpha.

CMSCLC displayed other stem cell characteristics, including the ability to form CFUs and be expanded in vitro. All CMSCLC cultures had similar potential with regard to cell doubling rates, while rates for CS-CDCs have been reported to be similar for cells generated from both nontransplant heart failure and transplant heart patient's biopsy specimens [26]. Under cardiac differentiation culture conditions, both CS-CDC and CMSCLC had a phenotype previously reported for cardiomyocytes in culture [28]. Moreover, in these conditions, all CS-CDC cultures, but only one CMSCLC culture, also contained a population of cells that expressed nuclear NXK2.5 and had an immature rounded morphology. These characteristics have previously been suggested to be indicative of a more primitive or early cardiac committed cell [29] and may suggest that the CS-CDCs are more heterogeneous than the CMSCLC or that the CMSCLC are more mature. This is further supported by our quantification of cells expressing troponin C, showing all CMSCLC containing higher numbers of troponin C expressing cells than CS-CDCs. In addition, we also observed that undifferentiated and differentiated CMSCLC expressed genes important for myocardial lineage development, including MEF2C and GATA4 [30,31]. Atrial appendage-derived cardiac stem cells also express markers important for myocardial development and even postinduction of differentiation to a more mature cardiac lineage, they continue to express MEF2C and GATA4 [32]. It is recognized that for MSCs derived from patient tissue, patient to patient variability does exist [33], and this could be responsible for the difference in the presence/absence of the NXK2.5 expressing cells in the CMSCLC cultures.

If cardiac stem/progenitors are to be used as a cellular therapy or indicator of heart health, the level of cellular senescence needs to be determined. We examined CMSCLC derived from three different donors at passage 3 and observed the presence of between 5% to 15% senescent cells. Whether the presence of senescent cells within CMSCLC would prove to be a problem in utilizing these as a cellular therapy would need to be further explored. CSC expanded over ∼113 days have been shown to contain small percentages of senescent cells (however, the authors of this study did not provide data on % observed) and yet still deemed suitable for intracoronary perfusion in patients with heart failure [1].

Human cardiac atrial appendage stem cells (CASCs), like CMSCLC, also fail to differentiate toward the osteogenic lineage. However, unlike the CMSCLC, they also failed to differentiate toward the adipogenic lineage [34]. CASCs also lack expression of c-kit and CD44 [34], while most of our CMSCLC cultures contain small numbers of c-kit+ cells and express CD44. It is interesting to note that both atrial appendages can be used as a source of stem cells. Why the atrial appendages are such good sources of stem cells is not entirely understood, but they have a distinct developmental origin to the atria and display many trabeculae similar to ventricles [10]. They are also a source of atrial natriuretic peptide factor (ANP). Nppa, the gene encoding ANP, has been shown to be important for heart development as well as being reactivated in adult disease [35]. It may be that these developmental/fetal links make the appendages such rich sources of stem cells. A study of mouse left atrial appendage showed it to be a source of more than one type of cardiac progenitor population [36].

As a cellular therapeutic, RAA-derived CMSCLC might have advantages over subendocardial-derived MSCs (that readily differentiate to osteogenic, adipogenic, and chondrogenic lineages, but remain primitive under cardiac differentiation conditions) and CS-CDCs or BM-MSCs as they lack the osteogenic potential of the former and the osteogenic and chondrogenic potential of the latter. This may have important implications for the use of CMSCLC as a potential cellular therapeutic as they could be less likely to contribute to cardiac calcification. The cause of this remains controversial, and while there is no evidence from clinical trials to date that cardiac-derived cell transplantation causes calcification, there is evidence that cells with osteogenic and chondrogenic differentiation potential may be involved [37]. CMSCLC also differ from CS-CDCs as they do not require the rounds of harvesting/replating required to generate CS-CDCs [25].

We did not observe spontaneous beating of CMSCLC under cardiac differentiation conditions; this may be a limitation of the technique used. Human CS-CDCs also failed to beat in vitro unless cocultured with neonatal rat ventricular myocytes [26], this was also the case with human CASCs [34].

Our single-cell analysis demonstrated that 100% of CMSCLC expressed CD44, a recognized marker of MSCs [19]. A large percentage of CMSCLC also expressed CDC73, which is associated with stem cell pluripotency [38]. Our single-cell analysis also supported our standard PCR analysis showing that GATA4 and MEF2 were also detectable at the single-cell level. GATA4 was expressed by all CMSCLC for all three donors (100%, 84.7%, and 82.5%), while MEF2C expression was more variable with two cultures having high numbers of MEF2C-positive cells (86.9% and 81.5%), but in the third only 31%. One hundred percentage of CMSCLC expressed tropomyosin and 98.5%, 71.7%, and 55% of CMSCLC expressed PCNA and nearly all expressed VEGF. Lower numbers of CMSCLC expressed MCAM (also known as CD146) with percentages varying from the highest at 58.4% to the lowest at 27.5%. CD146 has been reported to be expressed by a subpopulation of human MSCs from a telomerized bone marrow-derived stromal cell line [39]. While in the heart, it has been reported to be a marker of perivascular mesenchymal precursor cells [40]. Together, these data suggest that CMSCLC are proliferative, pluripotent, and have some multilineage differentiation potential compared with other cells important in the heart. It also supports our in vitro culture observation of some CMSCLC being capable of expressing the endothelial cell marker CD34 under EC differentiation conditions. CMSCLC for all donors also expressed TGF-β. The role of TGF-β in the heart remains controversial; it has been reported to contribute to heart failure, but has also been suggested to be important for suppression of inflammation following myocardial infarction and been reported to be cardioprotective [41,42]. CMSCLC also expressed VEGF (97.8%–100%), FGF2 (56.6%, 62.0%, and 100%), and HGF (18.0%, 41.3%, and 89.1%), which have all been reported to be cardioprotective/cardiobeneficial [42–44]. We also demonstrated using CMSCLC-conditioned media that CMSCLC secrete a number of potentially cardiobeneficial factors, including IL10, VEGF, FGF2, and TGFβ1. The role of MSC paracrine-secreted factors that have protective/regenerative effects has now been demonstrated in several different tissues. In a rat neonatal hyperoxic lung injury model, human umbilical cord blood MSCs that secrete VEGF improved the survival rates on rat lung epithelial cells treated with hydrogen peroxide; to confirm this protective effect, these cells were then used in an in vivo model of hyperoxic injury where it was again shown that the VEGF secreting MSCs protected against hyperoxia [45]. In a rat model of liver regeneration, BM-MSCs alone or transfected with the VEGF gene were injected into the liver after a major hepatic resection; both groups of cells engrafted into the liver were they secreted a number of paracrine factors, including VEGF, FGF, TGFβ, and HGF, and in both cases, bile duct and liver hepatocyte proliferation occurred [46]. The paracrine activity of MSCs may be multimodal as they have also been reported to be able to cause cardiomyocyte proliferation, be immunomodulatory, and activate resident cardiac stem cells spared by cardiac injury [reviewed in Ref. 47]. In addition, single-cell analysis showed only a few CMSCLC expressed c-kit, data that support the low percentages of c-kit expressing cells observed in the CMSCLC using FACS. Single-cell profiling as a means of determining the paracrine activity of MSCs has previously been used successfully in a study on the role of MSCs in an infracted mouse heart, where the authors showed using a single-cell approach that MSCs transplanted into injured heart expressed paracrine factors in vivo [48].

We have shown that CMSCLC cultured under cardiac differentiation conditions display morphology and expressed some markers of more mature cardiac lineage-committed cells, even showing early striation formation. Similar changes have been observed in rat CDCs differentiated to a more mature cardiac lineage in vitro [49]. In addition, CMSCLC secrete cardiobeneficial factors and have low/no adipogenic, chondrogenic, or osteogenic differentiation potential, all qualities that should be advantageous for cells to be used therapeutically for cardiac regeneration.

## Supplementary Material

Supplemental data

Supplemental data

Supplemental data

Supplemental data

Supplemental data

Supplemental data
