# Supplementary figures and images for: Human Cardiac-Mesenchymal Stem Cell-Like Cells, a Novel Cell Population with Therapeutic Potential
Source: Stem Cells Dev. 2019 Apr 25;28(9):593–607. doi: 10.1089/scd.2018.0170 (PMC6486668; doi:10.1089/scd.2018.0170)

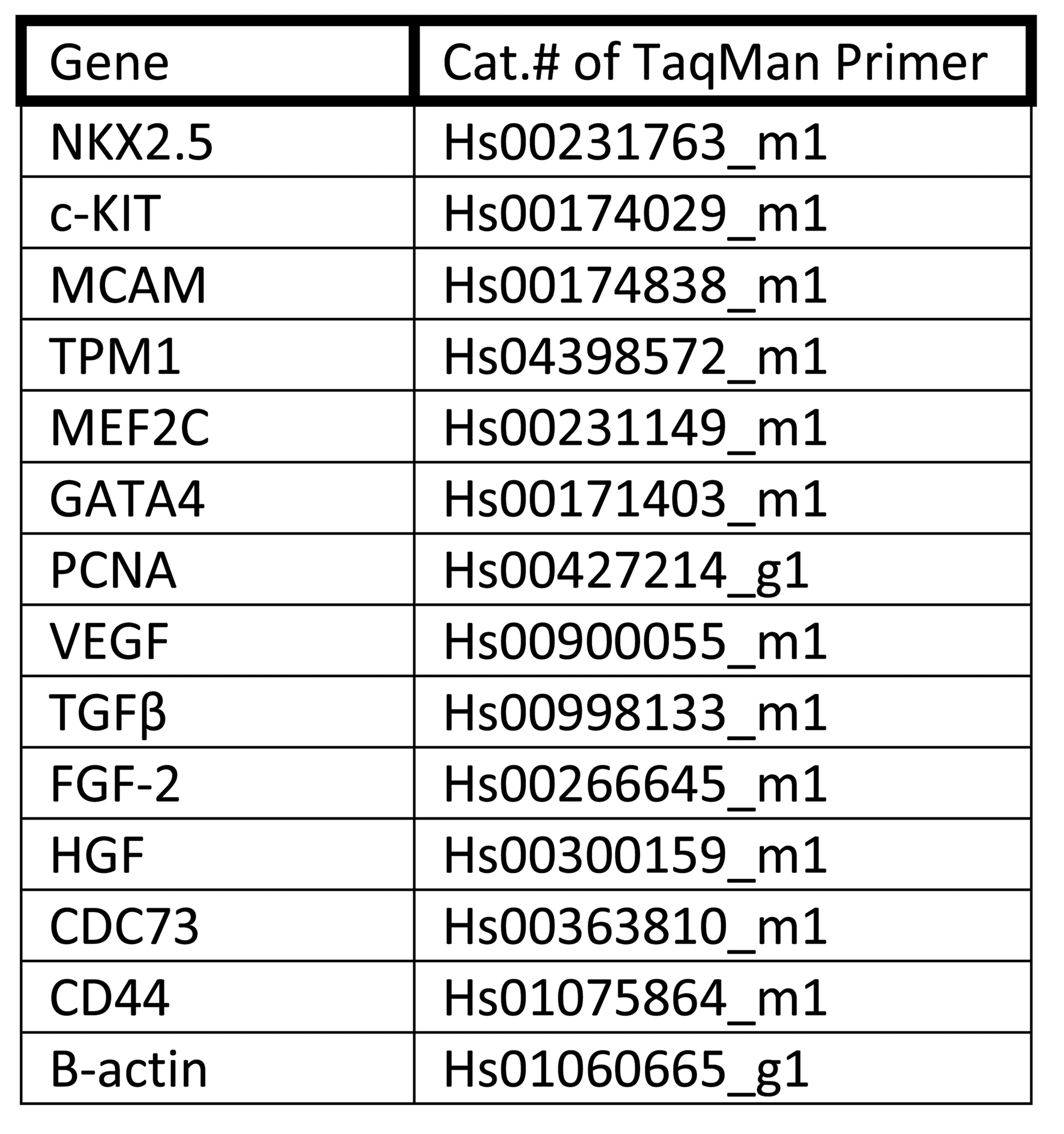

Supplement: Supplemental data [file Supp_Table1.tiff]
