# Supplementary material for: Human Cardiac-Mesenchymal Stem Cell-Like Cells, a Novel Cell Population with Therapeutic Potential
Source: Stem Cells Dev. 2019 Apr 25;28(9):593–607. doi: 10.1089/scd.2018.0170 (PMC6486668; doi:10.1089/scd.2018.0170)
Supplement: Supplemental data [file Supp_Fig1.pdf]

Supplementary Data

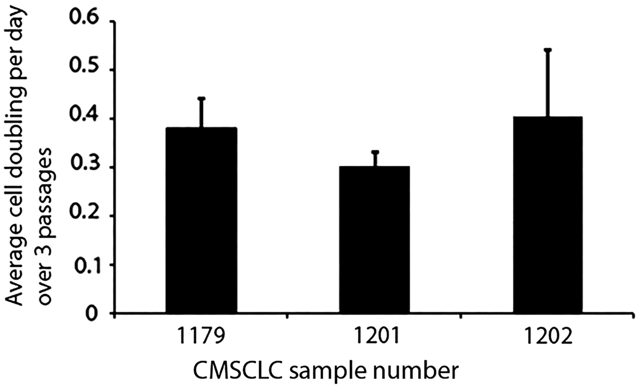

SUPPLEMENTARY FIG. S1. Analysis of doubling time of CMSCLC cultures. Cell population doublings per day were calculated for three patient-derived cultures, with values presented as the average rate of population doubling over three cell culture passages. No significant differences in cell doubling rates were observed between the three different cultures. CMSCLC, cardiac-mesenchymal stem cell-like cells.
