# Supplementary material for: Human Cardiac-Mesenchymal Stem Cell-Like Cells, a Novel Cell Population with Therapeutic Potential
Source: Stem Cells Dev. 2019 Apr 25;28(9):593–607. doi: 10.1089/scd.2018.0170 (PMC6486668; doi:10.1089/scd.2018.0170)
Supplement: Supplemental data [file Supp_Fig2.pdf]

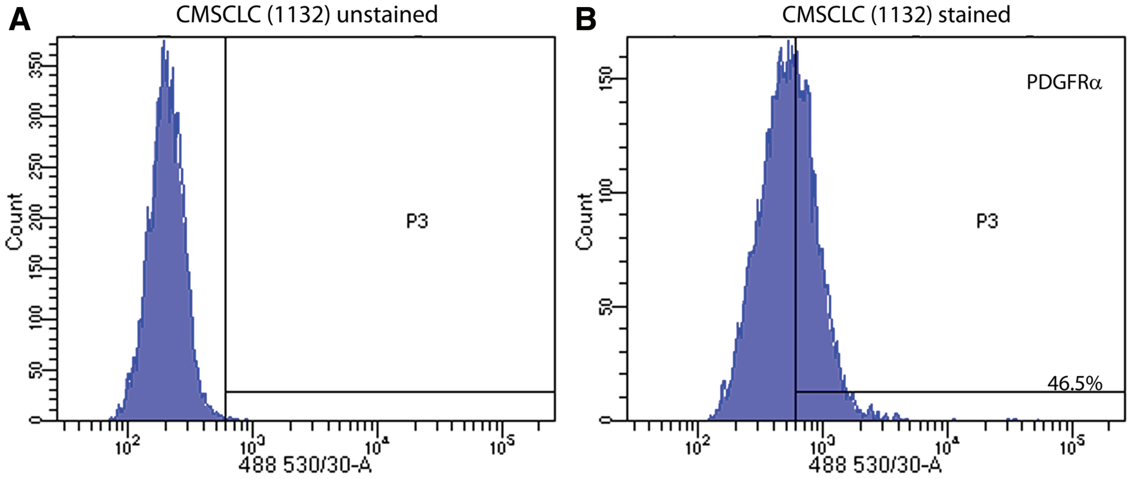

SUPPLEMENTARY FIG. S2. FACS analysis for expression of PDGFR-alpha by CMSCLC. Representative images of FACS histograms showing unstained CMSCLC **(A)** and cells stained for expression of PDGFR-alpha **(B)**. FACS, flow cytometry; PDGFR, platelet derived growth factor.
