# Supplementary material for: Human Cardiac-Mesenchymal Stem Cell-Like Cells, a Novel Cell Population with Therapeutic Potential
Source: Stem Cells Dev. 2019 Apr 25;28(9):593–607. doi: 10.1089/scd.2018.0170 (PMC6486668; doi:10.1089/scd.2018.0170)
Supplement: Supplemental data [file Supp_Fig3.pdf]

A

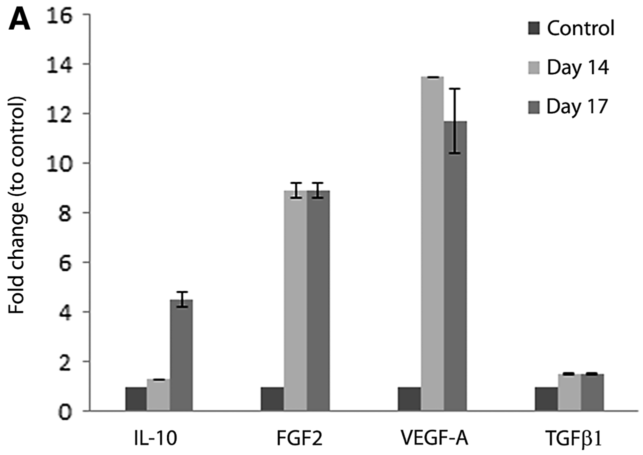

B

	IL-10	FGF2	VEGF-A	TGFβ1
Control - Day 14	NS	<0.0001	0.002	<0.0001
Control - Day 17	0.0005	<0.0001	0.001	<0.0001

SUPPLEMENTARY FIG. S3. Bar chart showing ELISA-detected fold changes in IL10, VEGF, FGF2, and TGFβ1 expression levels from CMSCLC cultured under standard CMSCLC conditions over time compared with control media (**A**). Note increased expression for all factors at day 14 and 17 compared with control. Note increases in fold change are significant for all factors at both time points with the exception of Il10 and 14 days, which were not significantly different (**B**).
