# Supplementary material for: Human Cardiac-Mesenchymal Stem Cell-Like Cells, a Novel Cell Population with Therapeutic Potential
Source: Stem Cells Dev. 2019 Apr 25;28(9):593–607. doi: 10.1089/scd.2018.0170 (PMC6486668; doi:10.1089/scd.2018.0170)
Supplement: Supplemental data [file Supp_Fig4.pdf]

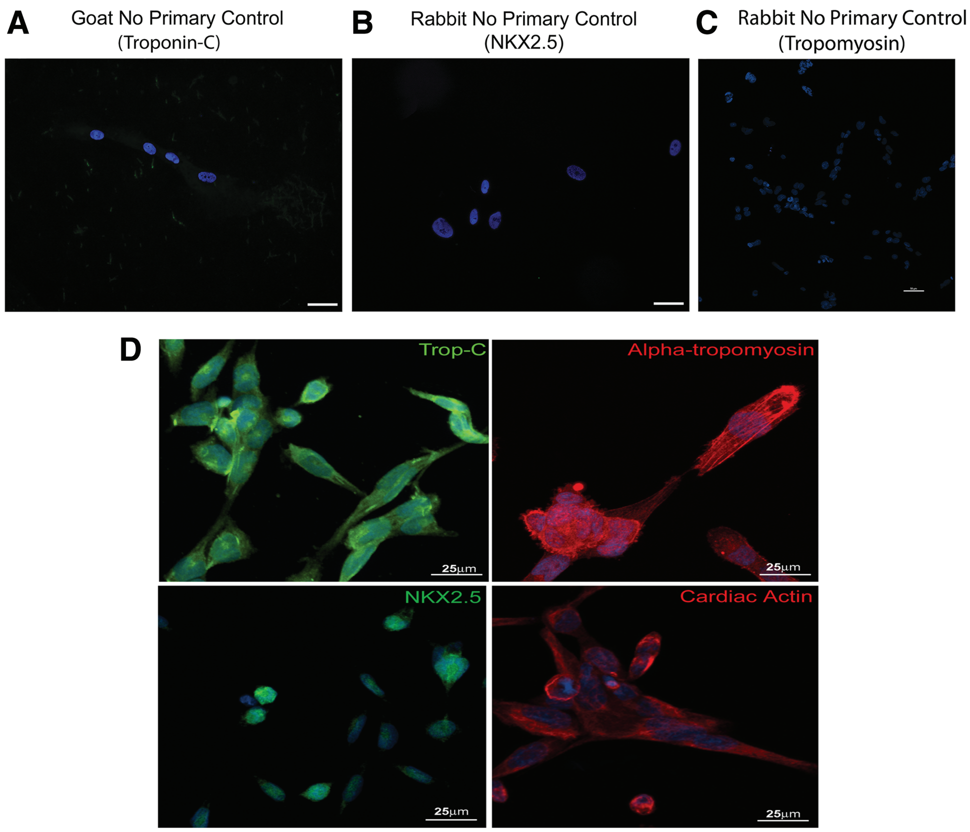

SUPPLEMENTARY FIG. S4. ICC controls for staining of CMSCLC. ICC analysis showing results of CMSCLC stained using secondary antibodies only (no primary) for troponin C, NKX2.5, and tropomyosin **(A)**. All nuclei are stained with DAPI (*blue*) (**A, B**), scale bars = 20 μm; **(C)**, scale bar = 50 μm. ICC analysis showing results of staining of the AC10 cell line **(D)** for expression of troponin C (*green*), alpha-tropomyosin (*red*), NXK 2.5 (*green*), and cardiac actin (*red*). All nuclei are stained with DAPI (*blue*). Scale bars = 25 μm.
