# Supplementary material for: Human Cardiac-Mesenchymal Stem Cell-Like Cells, a Novel Cell Population with Therapeutic Potential
Source: Stem Cells Dev. 2019 Apr 25;28(9):593–607. doi: 10.1089/scd.2018.0170 (PMC6486668; doi:10.1089/scd.2018.0170)
Supplement: Supplemental data [file Supp_Fig5.pdf]

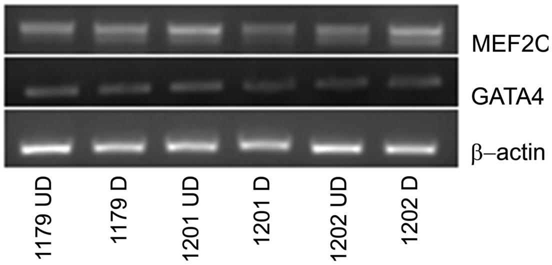

SUPPLEMENTARY FIG. S5. Transcriptional expression of MEF2C and GATA4 in undifferentiated and differentiated CMSCLC. Results of RT-PCR analysis of CMSCLC derived from three different patients. Note that expression of mRNA from both genes can be detected in both undifferentiated CMSCLC and cells from the same cultures after differentiation under cardiomyocyte differentiation conditions. β-actin was used as a loading control. Abbreviations: UD, undifferentiated; D, differentiated; RT-PCR, reverse transcription-polymerase chain reaction.

Supplementary Table S1. TaqMan Primers Used in Single-Cell Quantitative Polymerase Chain Reaction
